# Ergonomic evaluation of a mechanical anastomotic stapler used by Japanese surgeons

**DOI:** 10.1007/s00595-013-0666-6

**Published:** 2013-07-27

**Authors:** Emiko Kono, Mitsunori Tada, Makiko Kouchi, Yui Endo, Yasuko Tomizawa, Tomoko Matsuo, Sachiyo Nomura

**Affiliations:** 1 Department of Surgery, Osaka Kosei-Nenkin Hospital, Osaka, Japan; 2 Japan Association of Women Surgeons, Tokyo, Japan; 3 Digital Human Research Center, National Institute of Advanced Industrial Science and Technology, Tokyo, Japan; 4 Department of Cardiovascular Surgery, Tokyo Women’s Medical University, 8-1 Kawada Shinjuku, Tokyo, 162-8666 Japan; 5 Sales Planning and Project Management, Surgical Solutions, Covidien Japan, Tokyo, Japan; 6 Department of Gastrointestinal Surgery, Graduate School of Medicine, The University of Tokyo, Tokyo, Japan

**Keywords:** Ergonometric, Mechanical anastomotic stapler, Women Surgeons, Maximum grip force, Hand length

## Abstract

**Purpose:**

The satisfaction rating of currently available mechanical staplers for Japanese surgeons with small hands is low. To identify the issue, we examined the relationship of hand dimensions and grip force with the operation force of a mechanical circular stapler.

**Methods:**

Hand dimensions and grip force were measured in 113 Japanese surgeons (52 men and 61 women). We then evaluated the relationship between grip width and the operation force required to push the lever of the stapler, at three points on the lever, using a digital force gauge.

**Results:**

The optimal grip width of the dominant hand was 62.5 ± 8.5 mm for men and 55.5 ± 5.9 mm for women (*p* < 0.001). The maximum grip force of the dominant hand was 44.2 ± 6.1 kg for men and 29.7 ± 4.5 kg for women (*p* < 0.001) and the maximum operation force required to push the lever 7.0, 45.0, and 73.0 mm from the end of the lever was 21.8, 28.6, and 42.4 kg, respectively.

**Conclusions:**

To our knowledge, this is the first ergonomic study of a surgical stapler to be conducted in Asia. Firing the stapler by gripping the proximal side of the lever is physically impossible for most Japanese women surgeons since the required operation force exceeds the maximum grip force, which probably accounts for the stress perceived by these women.

## Introduction

In 1958, Professor Mine [1] developed and introduced the concept of a mechanical circular stapler in Japan. This stapler was used clinically. Later, Androsovn [2] in Russia simplified the mechanism of the stapler and developed the “PSK-25”, known as the “Suture Gun”. Most currently available mechanical anastomotic staplers are made in the United States and Europe. Among the mechanical anastomotic staplers, DST-EEA (Covidien, Mansfield, MA, USA) and CDH (Ethicon Co. Ethicon Endo-Surgery, Cincinnati, Ohio, USA) do not have handle size variation. This creates an ergonomic issue for Japanese surgeons who have relatively smaller hand dimensions and weaker grip force than surgeons from North American and European countries.

A survey on the ergonomic satisfaction of the currently available mechanical staplers among members of the Japanese Society of Gastroenterological Surgery (JSGS) clearly demonstrated that Japanese surgeons with small hands experience difficulties with using the staplers [3]. To identify the problems, we examined the relationship of hand dimensions and grip force with the operation force required to operate the mechanical stapler.

## Materials and methods

### Subjects

During the 2 days of the 66th General Meeting of the Japanese Society of Gastroenterological Surgery held in July 2011 in Nagoya, all the men and women surgeons who were in the conference hall were asked to participate in this study. The volunteers who agreed to participate were given a brief explanation of the objectives and procedures and all signed an informed consent form prior to the experiment. A total of 113 volunteers (52 men and 61 women) participated in this study.

Background information on the subjects, including gender, age, height, weight, and dominant hand, was collected by a self-report questionnaire. The average ages of the men and the women were 38.9 ± 9.3 and 33.4 ± 6.0 years, respectively. Means of the self-reported height and weight were 173.3 cm and 70.5 kg for the men and 159.3 cm and 52.8 kg for the women, respectively. Ethical approval for this study was obtained from the ethics committee of the Japanese Society of Gastroenterological Surgery on July 5, 2011.

### Measurement of hand dimensions

**Fig. 1:**
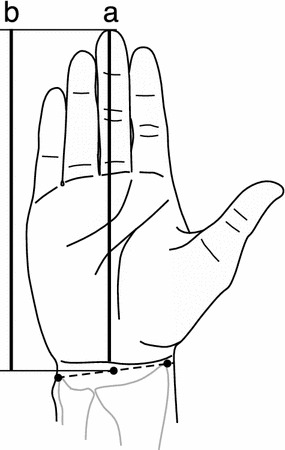
Definitions of hand length measurements. **a** hand length from the wrist crease, **b** hand length from the styloid processes

We measured the hand length from the wrist crease in 95 subjects, using a digital caliper (CDC-P20PMX, Mitutoyo Co., Kanagawa) with the tips of the jaws modified for measuring living people. The hand length of the remaining 18 men was measured from an image of the right palm scanned with a flatbed scanner (GT7300U, Seiko Epson Corp., Nagano) and in-house image processing software (Hand Matrix, Digital Human Research Center, Tokyo) retrieved the hand dimensions from the palmar image. The distance from the center of the most distal wrist crease to the tip of the middle finger was measured as the hand length for the present study (Fig. 1a).

The significance of gender differences in hand dimensions was tested using a *t* test with a significance level of 5 %. Statistical software (StatView Ver.5.0, Abacus Corporation, California) was used for this purpose.

### Measurement of grip force

Five grip dynamometers (T.K.K5401; Takei Scientific Instruments Co., Ltd. Tokyo) were used for measuring the grip force at various grip widths. The grip widths of the dynamometers were set at 40.0, 47.5, 55.0, 62.5, and 70.0 mm. For women, the grip force was measured from the narrowest grip width in ascending order. For men, the grip force was measured in the reverse order, from the widest to the narrowest. For both women and men, we measured the right hand first, and then the left hand. The optimal grip width and the maximum grip force were computed from these measurements.

### Measurement of operation force

**Fig. 2:**
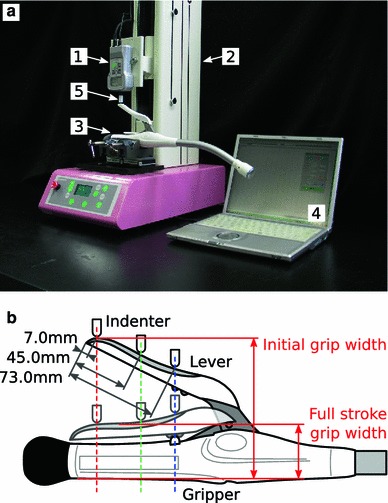
View of the system for measurement of the operation force (**a**) and schematic presentation of the pushing points on the lever (**b**). **a** *1* digital force gauge, *2* vertical-type motorized test stand, *3* mechanical anastomotic stapler, *4* host PC for recording, *5* indenter. **b** Three different pushing points (7.0, 45.0 and 73.0 mm from the end of the lever) on the lever, and positions of the lever in the initial and full stroke conditions

The operation force was measured at the Digital Human Research Center, National Institute of Advanced Industrial Science and Technology, Japan, using a digital force gauge (FGPX-50, Nidec-Shimpo Co., Kyoto) and a vertical-type motorized test stand (FGS-100VC, Nidec-Shimpo Co., Kyoto; Fig. 2a). The gripper of a mechanical anastomotic circular stapler (DST-EEA 28, Covidien, Mansfield, MA, USA) was clamped in two vices and fixed to the base of the stand so that the longitudinal axis of the gripper was parallel and the working plane of the lever was perpendicular to the base. Although the lever normally faces downward during surgery, the stapler was positioned with the lever facing upward in this measurement in order to simplify the experimental setup.

A cylindrical indenter, 10.0 mm in diameter and 20.0 mm in height, was attached to the detector of the force gauge. The indenter was made of engineering plastic (Delrin, Du Pont, DE, USA) and a fillet, 5.0 mm in radius, was introduced in the bottom face of the indenter to minimize friction between the lever and the indenter. The indenter pushed the lever at three different points: at both ends of the anti-slip rubber (approximately 7.0 and 73.0 mm from the end of the lever) and at the mid-point of the anti-slip rubber (approximately 45.0 mm from the end of the lever), at 100.0 mm/min until the lever reached the full stroke position. Since the lever turned around at the axis of rotation, the contact point between the indenter and the lever slid closer to the axis as the indentation depth increased (Fig. 2b). The sliding distances were approximately 25.0, 20.0, and 15.0 mm when the initial contact point was 7.0, 45.0, and 73.0 mm, respectively, from the end of the lever. The initial grip width was wider when the pushing point was closer to the end of the lever, while the full stroke grip width was wider when the pushing point was closer to the pivot of the lever (Fig. 2b).

To test the operation force with or without staples, two transparent silicone rubber sheets, 1.0-mm thick, were used as a test sample for measurement, since it is a homogeneous solid material with good reproducibility, and for visual confirmation of successful staple firing and fastening by the staples. After the initial measurement, additional measurements were repeated five times using the same device without staples and sheets (blank shot), to test for reproducibility. The indentation depth of the indenter and corresponding pushing force (operation force) were sampled at 50 Hz and transferred to an Excel sheet running on a host computer (Let’s Note CF-Y2DW1AXR, Panasonic Co., Osaka) through the USB port using an Excel plug-in (FGT-VC, Nidec-Shimpo Co., Kyoto).

### Optimal grip width and maximum grip force

The optimal grip width was defined as the grip width at which the maximum grip force was achieved. From the grip force measured under five different grip width conditions, the optimal grip width and the maximum grip force of each individual were calculated using a method proposed by Ruiz et al. [4–6]. For this purpose, a second-order polynomial regression curve was computed for each individual using the grip width as the independent variable and the grip force as the dependent variable.

If the second-order polynomial curve was a convex upward function and the local maximum was within the range of 40.0–70.0 mm, the maximum grip force was taken as the local maximum of the regression curve, and the optimal grip width as the grip width at maximum grip force. If either the local maximum was out of the above range or the regression curve was a convex downward function, then the maximum grip force was taken as the highest value of the five grip force measurements and the optimal grip width, as the corresponding grip width.

The significance of gender and laterality differences in optimal grip width and maximum grip force was tested using a *t* test at a significance level of 5 %. Statistical software (SciPy Ver. 0.10, Enthought Inc., TX, USA) was used for this purpose.

### Mean and 95 % confidence interval of the grip force

The mean and standard deviation of the grip force for each grip width were computed from the measured grip force of the dominant hand of the men and women surgeons. The 95 % confidence intervals were then obtained from the computed mean and standard deviation for each grip width. To examine the relationship between grip width and grip force, second-order polynomial regression curves were computed for the lower and upper limits of the 95 % confidence interval for both genders, using the grip width as the independent variable and the grip force as the dependent variable.

### Grip width and operation force of the mechanical anastomotic stapler

The relationship between grip width and the operation force required for operating the mechanical anastomotic stapler at each pushing point was evaluated by subtracting the measured indentation depth at each time step from the initial grip width. To evaluate the possible relationship between grip width and the operation force required, the lower and upper limits of the operation force were computed from the curves representing the relationship between the grip width and the operation force.

## Results

### Hand dimensions

**Fig. 3:**
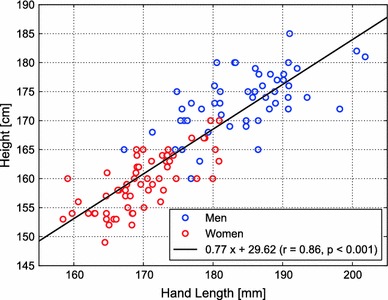
Relationship between hand length and the height of the surgeon. The *solid line* is a linear regression line based on all data

All subjects completed the questionnaire and measurements satisfactorily. The self-reported heights (mean ± SD) and weights were 173.0 ± 5.3 and 159.3 ± 4.8 cm and 69.8 ± 7.1 and 52.8 ± 7.6 kg for the men and women, respectively (both *p* < 0.001). The hand length was 184.1 ± 7.0 mm for the men and 169.5 ± 6.4 mm for the women (*p* < 0.001). There was a significant correlation between hand length and height for the combined data of the men and women (*r* = 0.86, *p* < 0.001; Fig. 3).

### Optimal grip width, maximum grip force, and 95 % confidence interval of grip force

**Fig. 4:**
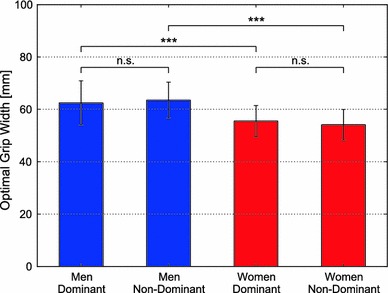
Comparison of the mean and standard deviation of optimal grip widths of the dominant and non-dominant hands of men and women. Result of *t* test: ***p < 0.001, *ns* not significant

Measurements were completed satisfactorily for all the subjects. The optimal grip width (mean ± SD) was 62.5 ± 8.5 mm for the dominant hand of the men, 63.5 ± 6.9 mm for the non-dominant hand of the men, 55.5 ± 5.9 mm for the dominant hand of the women, and 54.1 ± 5.9 mm for the non-dominant hand of the women (Fig. 4). The men had wider optimal grip width than the women for both the dominant and non-dominant hands (*p* < 0.001).

**Fig. 5:**
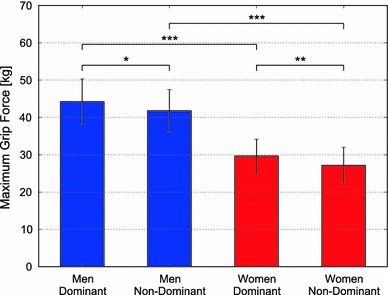
Comparison of the mean and standard deviation of the maximum grip forces of the dominant and non-dominant hands of men and women. Result of *t* test: *p < 0.05, **p < 0.01, ***p < 0.001

The maximum grip force (mean ± SD) was 44.2 ± 6.1 kg for the dominant hand of the men, 41.8 ± 5.7 kg for the non-dominant hand of the men, 29.7 ± 4.5 kg for the dominant hand of the women, and 27.2 ± 4.8 kg for the non-dominant hand of the women (Fig. 5). The men had greater maximum grip force than the women, for both the dominant and non-dominant hands (*p* < 0.001). Furthermore, the dominant hand had greater maximum grip force than the non-dominant hand for both the men (*p* < 0.05) and the women (*p* < 0.01).

**Fig. 6:**
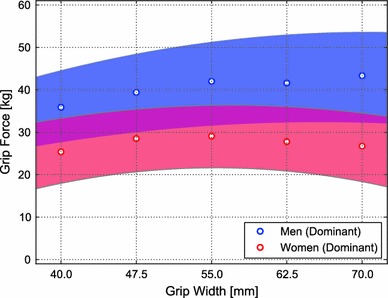
Mean (*circle*) and 95 % confidence interval (hatched region) of grip forces for men (*blue*) and women (*red*) (color figure online)

Figure 6 shows the 95 % confidence intervals of the grip force for the men and women at different grip widths. Grip forces were greater in the men than in the women for all grip widths (*p* < 0.001; data not shown). For each grip width, the average grip force for the men was greater than the upper limit of the 95 % confidence interval of the grip force for the women.

### Operation force

**Fig. 7:**
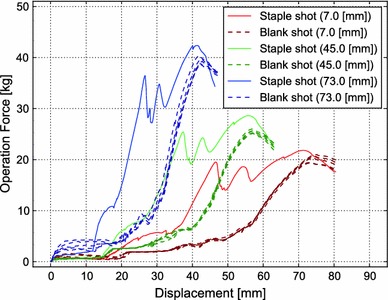
Relationship between displacement of the indenter and operation force at three different pushing points (7.0, 45.0 and 73.0 mm from the end of the lever), showing the operation force with (*solid line*, one measurement) and without (*dashed lines*, 5 measurements) staples

At all pushing points, the silicone sheets were successfully punched and stapled by the mechanical anastomotic stapler, as confirmed by visual inspection. Figure 7 shows the relationship between the displacement of the indenter and operation force at three different pushing points. With staples, the curves of the operation force showed three significant peaks from mechanical interactions among the staples, punch, and the anvil head. However, under (blank shot), no peaks were observed. The magnitudes (operation force) and the horizontal positions (displacement distance) of the peaks differed depending on the pushing points.

**Fig. 8:**
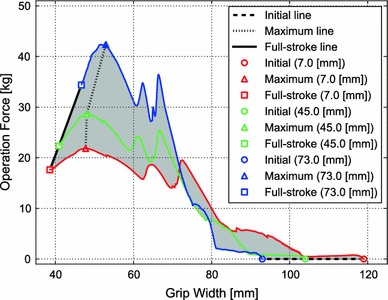
Relationship between the grip width and the operation force at the three different pushing points (7.0, 45.0 and 73.0 mm from the end of the lever)

As the pushing point approached the axis, the initial grip width became narrower (119.0, 104.0, and 93.0 mm for the 7.0-, 45.0-, and 73.0-mm pushing points, respectively), while the grip width at full stroke became wider (38.6, 40.9, and 46.6 mm, respectively; Fig. 2b). Also, as the pushing point approached the axis, both the maximum and full stroke operation forces increased. The line connecting the peaks was defined as the maximum line (Fig. 8, triangles), being 21.8, 28.6, and 42.4 kg for the 7.0-, 45.0-, and 73.0-mm pushing points. The differences in magnitude of the operation force as shown by the shaded area enclosed by the curves provide the upper and lower limits of the operation force (Fig. 8).

When an anastomotic device is used clinically, the hand comes in contact with the surface of the lever; however, when the hand exerts pressure on the lever, the center of pressure distribution can be regarded as the center of the operation force. The relationship between the grip width and operation force is thus approximated by a trajectory within the shaded area, starting from a point on the initial line (broken line), passing a point on the maximum line (dash line), then ending at a point on the full stroke line (solid line; Fig. 8).

## Discussion

### Problems and possible solutions

To our knowledge, this is the first Asian ergonomic study on a disposable mechanical anastomotic stapler imported from the United States. During gastroenterological surgery, surgeons are required to perform anastomosis successfully and securely, but this procedure is perceived to be stressful for surgeons with small hands, especially women [3]. This study is significant because the number of women surgeons in Japan is increasing [7, 8] and the physical characteristics of the hands of men and women differ: women have shorter hand length, narrower optimal grip width, and lower maximum grip force than men.

**Fig. 9:**
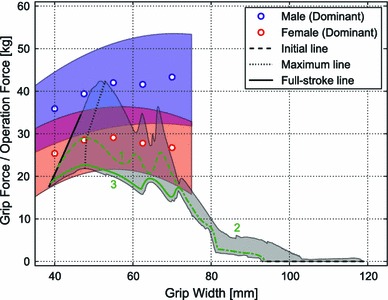
Relationship between the grip force of subjects and the operation force required to push the lever with three schematic trajectories. *1* The posterior half of the trajectory was lower than the lowest grip force of the men. *2* This *line* shows the anterior half of the trajectory with the grip width narrowed for small hands. *3* This *line* shows the posterior half of the trajectory for firing the anastomotic device with less operation force

The operation force required to push the lever to full stroke position differs according on the pushing point, since the lever has a rotation axis, and the length of the arm (distance from the rotation axis to the pushing point) helps to reduce the operation force required; however, it increases the total distance to move from the initial position to the full stroke position. A combination of these factors yields high and low limits of the operation force. The relationship between the grip force of the subjects and the operation force required to push the lever shows why there is ergonomic incongruity between the hand capability of Japanese women surgeons and the device properties (Fig. 9). For men surgeons, only the upper half of the maximum lies within the 95 % confidence interval of the grip force for men, and the lower half of the maximum line is below the lower limit of grip force. Men even with the weakest grip force are able to fire the anastomotic device because the trajectories of operation force when the lever is pushed in the middle (trajectory1 in Fig. 9) and at the distal end of the lever (trajectory 3 in Fig. 9) are below the lower limit of their grip force. Conversely, for women, the upper part of the maximum line extends beyond the upper limit of the 95 % confidence interval of the grip force for women, and the lowest point of the maximum line is greater than the lower limit of their grip force.

Some women surgeons have learned from experience to grip the proximal end of the lever with the non-dominant hand during the first half of the operation (trajectory 2 in Fig. 9) and then gradually move the dominant hand to grip the distal end of the lever (trajectory 3 in Fig. 9) in the latter half of the operation. These maneuvers effectively result in a trajectory following the lower limit of the operation force. The most important implication from Fig. 9 is that women surgeons with the lowest grip force are not capable of operating the mechanical anastomotic device with only their dominant hand even with this strategy, since the lowest operation force is greater than the lower limit of the 95 % confidence interval from around the maximum point to the full stroke point. Moreover, since the 95 % confidence interval of the grip force is lower in women than in men, the safety margin of the grip force against the operation force is lower in women, limiting their confidence in operating mechanical anastomotic devices. This may be the main reason for the stress felt by women surgeons when operating the mechanical stapler.

### Global variations in hand size

The statistics of hand length are listed in ISO 7250-2 (2010) [9]. In this document, hand length is defined as the distance from a line connecting the radial stylion (the most distal point of the styloid process of the radius) and the ulnar stylion (the most distal point of the styloid process of the ulna) on the palmar side to the tip of the middle finger (Fig. 1b). The average hand length was 189.6 mm for Japanese men and 175.4 mm for Japanese women vs. 202.0 mm for American men and 181.7 mm for American women [9]. The mean hand length for the Japanese people documented in ISO 7250-2 is longer than that in the present study, due to the difference in definition of hand length as the line connecting the stylion and ulnar stylion is more proximal than the most distal wrist crease used in this study.

The coefficient of correlation between hand length and height is influenced by the definition of hand length, body height, and the study population. According to Japanese data on young adults, based on measurements by experts, the coefficients of correlation ranged from 0.70 to 0.75. In the present paper, correlation was computed from the measured hand length and self-reported height, with resulting coefficients of correlation ranging from 0.64 to 0.69. Khanapurkar and Radke [10] reported that the coefficient of correlations for young Indian people aged 19- to 22-year old were 0.62 for men and 0.65 for women. All these data show relatively high positive correlations; therefore, we speculate that this tendency holds at least for Asian populations. The hand length of Japanese people is shorter than that of American people. According to the investigation of Berguer and Hreljac [11], the glove size is 7.5 or larger for 23.5 % of men surgeons, and 7 or larger for 19 % of women surgeons in the United States. On the other hand, only 1 % of women surgeons in Japan wear gloves of size 7 or larger, whereas 99 % wear gloves of size 6.5 or smaller [3]. The average grip force was reported to be 62.2 kg for the dominant hand and 58.5 kg for the non-dominant hand of American men surgeons, and 36.7 kg for the dominant hand and 34.4 kg for the non-dominant hand of women surgeons [12]. The hand sizes in other Asian countries are close to those in Japan. The mean hand length is 185.8 and 174.7 mm in South Korean men and women [9], respectively, and 183 and 171 mm in Chinese men and women, respectively [13]. Considering the similarity in hand dimensions, the problems in handling anastomotic staplers experienced by Japanese surgeons are also likely to be experienced by surgeons in other Asian countries. Ultimately, all surgeons should be able to operate these surgical devices easily and comfortably regardless of hand dimension and grip force. Thus, surgical devices should be developed based on the guidelines of ergonomics [14–17].

### Limitations of the study

This study has some limitations: first, the ratio of the male and female subjects was not identical to the actual gender ratio in the Japanese clinical situation, since we intentionally recruited women surgeons; second, the maximum grip force may have been over-evaluated because the hand dynamometer was easier to grasp than the stapler in the clinical setting; third, the firing force of the mechanical anastomotic stapler may have been under- or over-estimated using silicone sheets as test material because of the difference in resistance between silicone sheets and fresh gut tissues; and fourth, only one product from one company was examined. A similar experiment is necessary to examine multiple devices from other companies.

## Conclusions

Women surgeons have shorter hand length and weaker grip force than men surgeons in Japan. Thus, firing the circular stapler by gripping the proximal side of the lever is physically impossible for most of Japanese women surgeons since the required operation force exceeds the maximum grip force for a given grip width.
